# Endoscopic Sclerotherapy with a Large Volume of High Concentration of Cyanoacrylate for Jejunal Variceal Bleeding bys Single-Balloon Enteroscopy

**DOI:** 10.3390/medicina54050068

**Published:** 2018-10-09

**Authors:** Jyong-Hong Lee, Chih-Sheng Wu, Jen-Hsuan Huang

**Affiliations:** 1 Digestive Disease Center, Department of Medical Research, Show-Chwan Memorial Hospital, Changhua 500, Taiwan; huanghuatzu@gmail.com (J.-H.L.); wuusevet@show.org.tw (C.-S.W.); 2 Department of Anesthesiology, Show-Chwan Memorial Hospital, Changhua 500, Taiwan

**Keywords:** jejunal varices, sclerotherapy, single-balloon enteroscopy

## Abstract

Jejunal varices are a rare manifestation of portal hypertension, and they are associated with a high mortality and poor prognosis when bleeding occurs. A bleeding jejunal varix is much more challenging to diagnose and manage because of its anatomic location. Herein, we describe the case of a 62-year-old man with active jejunal variceal bleeding who presented with massive hematochezia and hypovolemic shock. He was treated successfully with a high volume and concentration of a glue mixture as endoscopic sclerotherapy using single-balloon enteroscopy in the intensive care unit. Enteroscopic sclerotherapy is an effective option for jejunal variceal bleeding.

## 1. Introduction

Ectopic varices, defined as varices other than those in the esophagus and stomach, are relatively uncommon [1], and small intestinal varices account for 6.4% of ectopic varices [2]. Bleeding from jejunal varices is generally massive, and the associated mortality rate is greater than 40% [3]. Because of the anatomic location of jejunal varices compared with esophageal or gastric varices, jejunal varices bleeding is much more difficult to detect and manage [4]. Hence, successfully treatment has been rarely reported [5,6,7,8]. Although surgery is the first choice for managing active bleeding traditionally, it is invasive and risky, especially in patients with cirrhosis and poor hepatic function [7,8]. Single-balloon enteroscopy (SBE) is designed to identify small intestinal lesions, and it has been reported as an efficient diagnostic and therapeutic tool for treating of bleeding due to small intestinal vascular lesions [9]. We describe a case of active jejunal varices bleeding that was successfully treated with sclerotherapy using SBE facilitated with general anesthesia in the intensive care unit.

Study is approved by the Institutional Review Board of Show Chwan Memorial Hospital (SCMH_IRB No: 1070903, approved 10/8/2018). 

## 2. Case Report

A 62-year-old man weighing 76 kg experienced melena for two days. He also had alcoholic liver cirrhosis complicated by hepatocellular carcinoma (HCC) (T2N0M0, Barcelona-Clinic Liver Cancer stage A) and he had undergone transcatheter arterial embolization (TAE) and laparoscopic radiofrequency ablation. He also had esophageal varices and gastric varices, and received endoscopic variceal band ligation due to bleeding esophageal variceal bleeding. He smoked 20 cigarettes per day and drank an average of 100 g alcohol daily for about 40 years without quitting. On admission, his blood pressure was 116/74 mmHg, pulse rate was 80 beats/min, respiratory rate was 20 breaths/min, and body temperature was 35.9 °C. The hemoglobin level decreased to 8.3 g/dL (baseline hemoglobin level: 9.2 g/dL). Abdominal computed tomography (CT) showed cirrhosis and recurrent HCC in S4, S6, and S7 with thrombosis in the right posterior branch of the portal vein.

**Figure 1:**
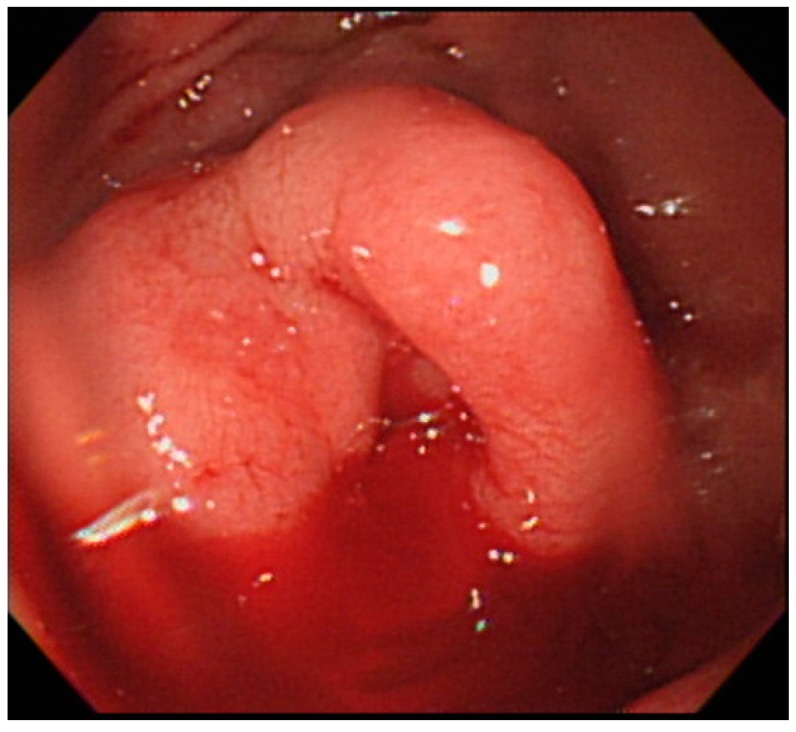
One engorged cystic lesion with persistent bleeding from the central depression observed in the proximal jejunum with single-balloon enteroscopy.

**Figure 2:**
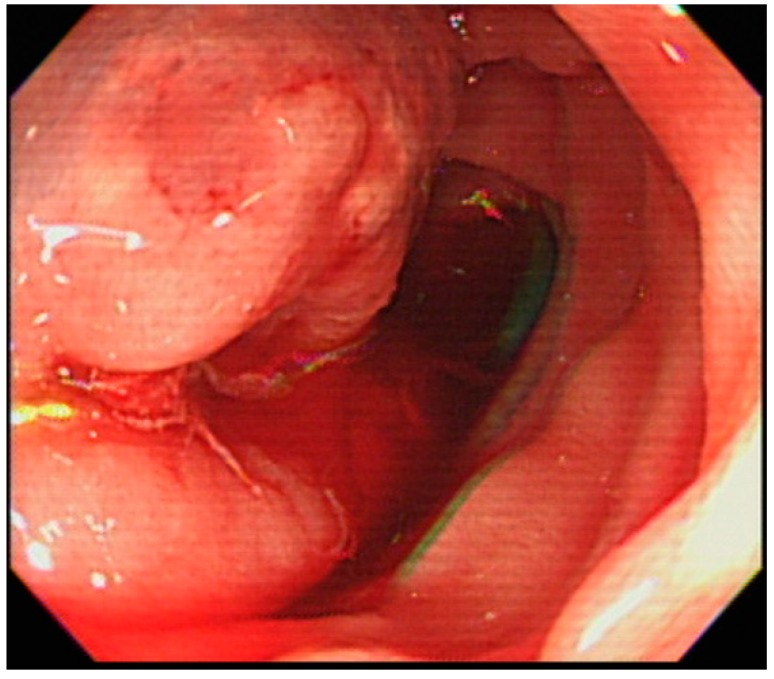
After the first attempt of sclerotherapy, jejunal variceal bleeding persists.

**Figure 3:**
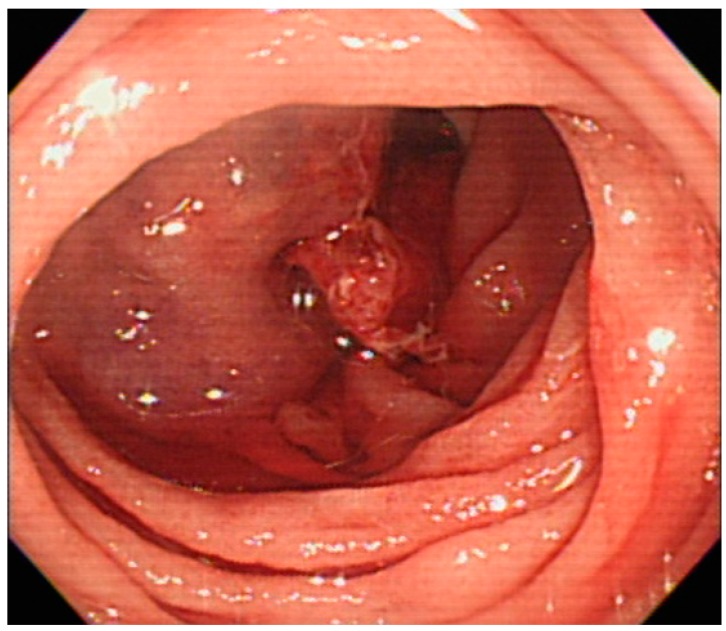
The sclerosant fills the varices and bleeding ceases after the second attempt of sclerotherapy.

He was transferred to the intensive care unit for hemorrhagic shock. Both emergent esophagogastrodudenoscopy (EGD) and colonoscopy failed to reveal the source of hemorrhage. Under the impression of massive obscure gastrointestinal bleeding and suspicion of small bowel bleeding, we decided to perform anterograde SBE (Olympus Medical systems, Tokyo, Japan). In the proximal jejunum, a large amount of fresh blood and 1 engorged cystic lesion with persistent bleeding from the central depression were observed (Figure 1). A soft sensation (not induration) was felt through the biopsy forceps, and ectopic jejunal varices were initially diagnosed. A 3.5 mL glue mixture (3.0 mL of cyanoacrylate and 0.5 mL of lipiodol) was injected into the varix. Because of persistent bleeding (Figure 2), a second attempt of sclerotherapy with 2.0 mL of cyanoacrylate was performed. The sclerosant filled the varices and bleeding ceased (Figure 3).

**Figure 4:**
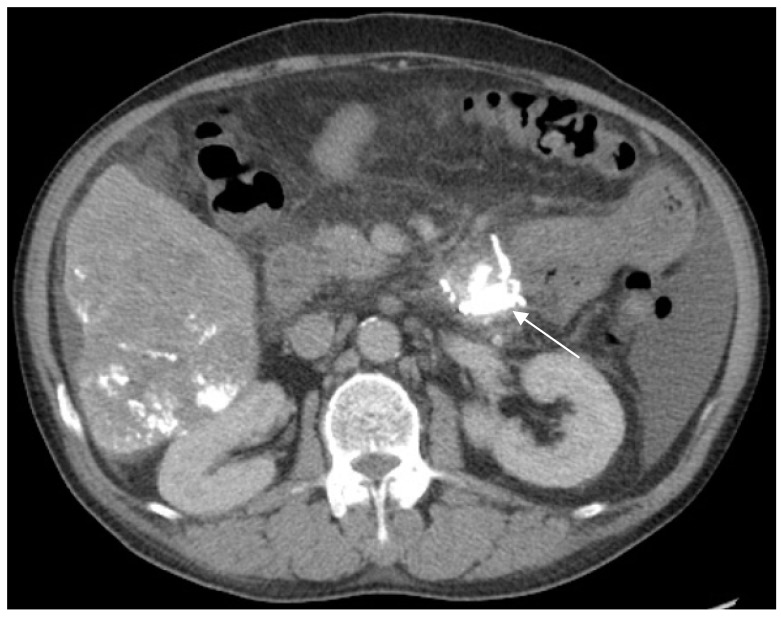
Abdominal contrast-enhanced computed tomography scan confirms the deposition of dense lipiodol in the proximal jejunum four days after sclerotherapy. The previous transcatheter arterial embolization caused some deposition of lipiodol in the liver, and the amount is unchanged from that observed in the prior image.

Four days later, abdominal contrast-enhanced CT confirmed the deposition of dense lipiodol in the proximal jejunum (Figure 4). Melena stopped after the procedures were completed, and no sclerotherapy-related complications occurred. The patient was discharged on admission day 15 uneventfully. He eventually died of underlying liver disease six months later without any bleeding diathesis.

## 3. Discussion

Varices bleeding due to portal hypertension is a main cause of cirrhosis-related morbidity and mortality. In patients with chronic liver disease and portal hypertension who experience obscure bleeding, which is defined as recurrent bleeding when the source remains unidentified after upper endoscopy and colonoscopic evaluation, jejunal variceal bleeding should be considered. Three clinical features of jejunal variceal bleeding have been described, including portal hypertension, a history of abdominal surgery, and hematochezia without hematemesis, which were consistent with our patient [10].

The predisposing risk factors for jejunal varices formation are hepatic cirrhosis with portal hypertension, HCC, pancreatitis, a history of abdominal surgery, nodular lymphoid hyperplasia, and familial small intestinal varices without portal hypertension [8]. Previous abdominal surgery can lead to the development of ectopic varices at the surgical site or cause postsurgical jejunal adhesion. Portal hypertension due to alcoholic cirrhosis, HCC, and a history of abdominal surgery (laparoscopic radiofrequency ablation for HCC) could explain the occurrence of jejunal varices in our patient.

It is impossible to detect jejunal varices by using conventional EGD and colonoscopy because of the depth, length, and tortuosity of the jejunum. It has been reported that capsule endoscopy is invaluable and highly sensitive for detecting small intestinal variceal bleeding [11]. Other diagnostic modalities such as CT enterography, nuclear scans, and magnetic resonance enterography could improve the diagnosis of small intestinal lesions but not therapy. SBE makes it possible to observe the jejunum, and it can be used for treatment, such as sclerotherapy or cauterization [12,13].

Due to acute hemorrhagic shock, the initial management is resuscitation with correction of dehydration, anemia, and coagulopathy in our case. Although the role in jejunal varices has not been estimated, somatostatin has been widely used in the management of esophagogastric variceal bleeding with the effect of decreasing portal blood inflow and variceal pressure [14] and may be beneficial in our patient.

Currently, the best treatment option for jejunal varices bleeding has not been proposed and verified. Because of the inaccessibility and difficult implementation of the treatment, the management of jejunal variceal bleeding is still considered challenging. The selection of possible therapeutic options is made considering the risks and benefits based on the site of the varices, patient’s condition, doctor’s experience, and the capability of the facility [15]. Therapeutic options for bleeding ectopic varices include local treatment for the varices and portal decompression [16]. Local treatment for the varices comprise endoscopic sclerotherapy, endoscopic variceal band ligation, endoclips, percutaneous transhepatic obliteration, and balloon-occluded retrograde transvenous obliteration. Portal decompressive treatment includes surgical dissection, surgical portosystemic shunting, and transjugular intrahepatic portosystemic shunting, which may result in a significant mortality rate, shunt occlusion, hepatic encephalopathy, and renal failure; these treatments should be performed by well-trained surgeons or radiologists.

Considering the risks and benefits in our case, we performed local treatment for the jejunal varices with endoscopic sclerotherapy using SBE after obtaining informed consent from the patient’s family. Endoscopic sclerotherapy has been used to treat bleeding duodenal varices and suggested as a practical and effective measure [17]; however, studies about jejunal varices are rare. SBE can be used examine the deep small intestine with the diagnostic and therapeutic approaches in cases of ectopic variceal bleeding [12].

The potential side effects of the endoscopic sclerotherapy for ectopic varices are perforation, tissue injury, embolism, and rebleeding from the varices or posttreatment ulcers [17]. Our choice of sclerosant is cyanoacrylate, which is a monomer-based tissue adhesive that instantly polymerizes and solidifies upon contact with the hemorrhage. Compared to other sclerosants, cyanoacrylate causes less tissue injury [17]. Unlike gastric varices, jejunal varices are the result of dilatation of the proximal branches of the superior mesenteric vein [6] that are within a short distance from the intrahepatic portal vein, and a higher volume of sclerosant may increase the risk of embolism [18] and obstruction of portal venous flow. Similar to Kitagawa Sho et al. [5], we used higher concentration of glue mixture (3.0 mL of cyanoacrylate and 0.5 mL lipiodol) than the usual concentration (ratio of cyanoacrylate to lipiodol, 1:1 or 1:1.6) with a 3.0 mL bolus of cyanoacrylate in the second attempt to shorten the polymerization time and to prevent leakage into the liver. Fortunately, no sclerotherapy-related complications occurred after the procedure in our case, and previous TAE-related lipiodol deposition in the liver was unchanged from that shown on the prior CT image. To our knowledge, we used a larger volume and higher concentration of glue mixture as endoscopic sclerotherapy to treat jejunal variceal bleeding than those used in previous case reports [5,15,19].

## 4. Conclusions

In conclusion, if jejunal varices bleeding is strongly suspected, SBE with sclerotherapy performed by an experienced endoscopist should be an alternative treatment instead of surgical or surgical intervention. Further studies are needed to establish the effectiveness and safety of this treatment for jejunal varices bleeding.
